# Development and Sensory Evaluation of Omega-3-Rich Nile Perch Fish Oil-Fortified Yogurt

**DOI:** 10.1155/2021/8838043

**Published:** 2021-02-15

**Authors:** Margaret W. Murage, Edward K. Muge, Betty N. Mbatia, Mercy W. Mwaniki

**Affiliations:** ^1^Department of Biochemistry, University of Nairobi, P.O. Box 30197 Nairobi, Kenya; ^2^Department of Biological Sciences, University of Kabianga, P.O. Box 2030-20200 Kericho, Kenya; ^3^School of Pharmacy and Health Sciences, United States International University-Africa (USIU-A), P.O. Box 14634-00800, Nairobi, Kenya; ^4^Department of Food Science and Technology, The Technical University of Kenya, P.O. Box 52428-00200, Nairobi, Kenya

## Abstract

Nile perch (*Lates niloticus*) is a major fish species in East Africa and its processing produces sufficient amounts of by-products containing significant amounts of long-chain polyunsaturated fatty acids (PUFAs). Due to the health benefits associated with PUFAs, they can be incorporated into commonly consumed foods such as yoghurt. This study is aimed at developing an omega-3-rich functional yoghurt and evaluating its quality and acceptability. Omega-3-rich fish oils were obtained from Nile perch fat pads in the presence and absence of a commercial food grade enzyme Alcalase. Recovery of omega-3-rich fish oil was done by centrifugation at 1000 × *g* at room temperature. The peroxide value (PV), anisidine value (AV), total oxidation (TOTOX), and free fatty acids (FFA) were some of the quality parameters investigated. Natural yoghurt (150 ml) was prepared and spiked with 3.5 g of omega-3-rich Nile perch oil. To mask the fishy flavor and taste, four different flavors were used and sensory evaluation of the yoghurt samples was performed. The liberation of Nile perch fish oil in the absence of Alcalase gave better yield (60.7% wet weight), while the use of Alcalase gave lower yields (48.3% wet weight). Assessment of the quality of the extracted fish oils showed that all parameters were within the required limits. Sensory characterization by a panel of students showed that passion and strawberry flavors were the most liked with mean values of 4.65 and 4.625, respectively. This study revealed that substantial amounts of omega-3-rich fish oil can be extracted from Nile perch fish pads in the absence of exogenous enzymes. Fortification of yoghurt with omega-3-rich Nile perch fish oils is an approach towards increasing omega-3 intake within the Kenyan population and globally.

## 1. Introduction

In recent years, increasing incidence of noncommunicable diseases (NCD) risk factors, including reduced physical activity, unhealthy diets, diabetes, and obesity among others, has led to a surge in the burden of NCDs [1]. Cardiovascular diseases (CVD) are examples of noncommunicable diseases whose rapid increase has been associated with decreased intake of omega-3 fatty acids [2]. Previous studies have reported that these omega-3 fatty acids reduce the risk of developing heart diseases [3]. Other studies show that omega-3 fatty acids are vital in the treatment of disorders such as Alzheimer's disease [4], help in the reduction of bone loss [5], prevent depression incidences, and among many other benefits [6, 7]. Two essential fatty acids, linoleic and alpha-linolenic, cannot be synthesized by humans; however, they are the main building blocks for omega-3 and omega-6 fatty acids [8]. Therefore, these fatty acids must be provided in the diet or through supplementation. In Kenya particularly, studies have reported a significant decrease in fish consumption which is attributed to the high cost of fish and omega-3 supplements as well as some traditional beliefs among some Kenyan communities [9]. It is therefore important to identify alternative methods that can increase the amount of omega-3 fatty acids provided in the diet among the Kenyan population. One strategy is through food fortification with omega-3-rich fish oils [10]. Nile perch (*Lates niloticus*) is a warm water fish present in East Africa lakes and rivers. Some of the by-products of Nile perch processing include the head and viscera which are reported to have sufficient amounts of omega-3 fatty acids [11, 12]. Other studies have reported that fish oils extracted from Lake Victoria Nile perch belly flaps are rich in omega-3 fatty acids [13, 14]. Besides, if these by-products are not properly utilized, they are likely to also be deposited in the environment causing pollution and other related health issues [13–15]. A previous study indicated that on average, every 100 g of the fat pads from viscera and belly flaps of Nile perch contains about 750 mg (7.5%) of omega-3-rich oils [16]. Fortification of commonly consumed foods with omega-3-rich fish oils, therefore, increases their availability in the diet. Yoghurt is commonly consumed especially by children and can be fortified with omega-3-rich fish oils [17]. Sensory analysis in food product development is commonly done to determine consumer preference and perception for a product [18]. In the current study, by-products produced by Nile perch fish processing were used in the preparation of flavored yoghurt fortified with omega-3-rich Nile perch fish oils.

## 2. Materials and Methods

Nile perch processing by-products were obtained from a Nile perch processing plant (W.E. Tilley Ltd., Kenya). Food grade enzyme Alcalase was purchased from Novozymes (Bagsvaerd, Denmark) and used to hydrolyze the fat pads. Starter cultures of *Lactobacillus bulgaricus* and *Streptococcus thermophilus* were locally purchased. Silica gel 60 TLC plates were purchased from Merck (Darmstadt, Germany). All the solvents and chemicals used were of analytical grade, including 0.1 N sodium hydroxide and hexane/diethyl ether/acetic acid (50 : 50 : 1, *v*/*v*/*v*) (Fisher Scientific, Fair Lawn, NJ) for TLC analysis; iodine and 0.2 ml saturated potassium iodide (Sigma-Aldrich Co., St. Louis, MO); 0.01 N sodium thiosulfate and glacial acetic acid (BDH Chemicals Ltd., Poole, England); chloroform and 99.8% 2,2,4-trimethylpentane (Sigma-Aldrich Chemie, Steinheim, Germany); and 99% p-anisidine, phenolphthalein, and ethanol (Sigma-Aldrich Co., St. Louis, MO).

### 2.1. Sample Preparation

Using a grinder (Sumeet Research and Holdings PVT Limited, Tamil Nadu, India), grinding and homogenization of 5 kg of Nile perch viscera was done at 4°C to reduce the particle size. The homogenates were stored at −20°C until further analysis.

### 2.2. Oil Extraction from Nile Perch Fat Pads

Enzymatic extraction of the omega-3-rich fish oils was done as described by Mbatia et al. [16] with slight modifications. The optimum temperature was adjusted to 55°C, and 2 ml of Alcalase per 100 g of the homogenized fat pads was used, followed by 1 h incubation. Inactivation of the enzyme was done by incubating the hydrolysates at 100°C for 20 minutes. Oil extraction was also performed similarly but without the Alcalase enzyme.

### 2.3. Recovery of Omega-3-Rich Oil from the Hydrolysate

To recover omega-3 fatty acid-rich oil, centrifugation (1000 × *g*, room temp) of the hydrolysate obtained in the presence and absence of Alcalase was performed to separate the oily fraction, emulsion, aqueous, and sludge phases. The wet weights of the different fractions were determined by placing the falcon tubes in an upright position at -20°C for 2 h. Separation of the fractions was achieved by cutting off the frozen tubes and determining the wet weights. The experiment was performed in triplicates, and the mean ± {standard deviation (SD)} of the wet weights was determined.

The oily fraction was decanted off into 50 ml centrifuge tubes (Thermo Fisher Scientific, Fair Lawn, NJ) and centrifuged (1000 × *g*, 4°C) for 20 minutes. A liquid oil (omega-3-rich oil) and solid fat fraction were obtained. The weight of the two fractions was determined and the upper liquid layer which was rich in omega-3 fatty acids used in yoghurt fortification.

### 2.4. Thin Layer Chromatography Analysis

Separation of the entire homogenate before centrifugation, the oil phase, and the emulsion was performed on silica gel 60 thin layer chromatography (TLC) plates (Merck, Darmstadt, Germany), with corn oil used as a standard. Elution was performed using a mobile phase comprising of hexane/diethyl ether/acetic acid (50 : 50 : 1, *v*/*v*/*v*). Iodine vapor was used to visualize the lipids, while identification of the bands was determined using the formula described by Mbatia et al. [16].

### 2.5. Quality Parameters of the Nile Perch Oil

#### 2.5.1. Peroxide Value (PV)

The official methods by the American Oil Chemists Society (AOCS) were used to determine the PV (meq/kg) [19]. Equation (1) was used where *S* denotes the volume (ml) of Na_2_S_2_O_3_ used in titrating the sample, *B* is the volume (ml) of Na_2_S_2_O_3_ used in titrating the blank, *M* is the molar concentration of Na_2_S_2_O_3_ (*N*), and *W* is the sample weight in grams.

(1) $$\begin{array}{c} \text{PV}=\frac{\left(S-B\right)\times N\times 1000}{W}. \end{array}$$

#### 2.5.2. Anisidine Value (AV)

To determine the AV [20], about 1 g (*M*) of the extracted fish oil was weighed and added into a 25 ml volumetric flask. Anhydrous (99.8%) 2,2,4-trimethylpentane (isooctane) was used to dissolve the oil to volume. The absorbance (*Ab*) was measured at 350 nm, and the solvent was used as a blank. An aliquot (5 ml) of the fat solution was pipetted, and an equal volume of the solvent was added. About 1 ml of 99% *p*-anisidine reagent (0.25 g/100 ml glacial acetic acid) (Sigma-Aldrich Co., St. Louis, MO) was added into each test tube with vigorous shaking. The absorbance (*As*) was measured at 350 nm (Thermo Scientific UV-Vis spectrophotometer, Germany), and the solution in the second test tube was used as a blank (*Ab*). AV value was determined using the following equation:

(2) $$\begin{array}{c} \text{AV}=\frac{25\;\left(1.2As-Ab\right)}{M}. \end{array}$$

#### 2.5.3. Total Oxidation (TOTOX)

Total oxidation (TOTOX) values were determined as described by Wai et al. [20] using the following equation:

(3) $$\begin{array}{c} \text{TOTOX}=2\text{PA}+\text{AV}, \end{array}$$

where PA is the peroxide value and AV is the anisidine value.

#### 2.5.4. Free Fatty Acid (FFA) Content

The titration method described by [17] was used to determine the FFA content (%). To 10 ml of heated and neutralized ethanol solution, 2 g of the extracted fish oil was added and mixed with 0.4 ml of phenolphthalein in a 125 ml flask. 0.1 N sodium hydroxide (NaOH) was used to titrate the mixture. FFA (%) was determined using the following equation:

(4) $$\begin{array}{c} \text{FFA}\left(\%\right)=\frac{\left(\text{NaOH}\left(\text{ml}\right)*N*28.2\right)}{\text{mass}\left(\text{g}\right)}, \end{array}$$

where *N* is the normality of the NaOH and mass (g) is the mass of sample used.

### 2.6. Development of Omega-3-Fortified Yoghurt

The method described by Mbatia et al. [16] was used to determine the amounts of omega-3-rich fish oil incorporated into the yoghurt. Based on this method, 100 g of Nile perch oil contains 20.5% of omega-3 fatty acids which includes 3% EPA, 6.2% DPA, and 9.0% DHA. From this inference, 3.5 g of the omega-3-rich oil phase was incorporated into 150 g of yoghurt. This resulted in approximately 0.7 g-1 g of omega-3 fatty acids per 150 g yoghurt. This is more than the average levels recommended by WHO of 500 mg/day of omega-3 fatty acids [18]. A functional fortified yoghurt was developed in our food laboratory as described in Table 1. To solubilize the fish oil in the yoghurt, skimmed milk powder with soy lecithin was used as a thickener and stabilizer.

### 2.7. Method of Yoghurt Fermentation

The yoghurt mix formulations were prepared in batches in glass containers. The homogenized mixtures were batch pasteurized at 85 ± 1°*C* for 30 min, rapidly cooled in an ice bath to 43 ± 1°*C*. Commercial freeze-dried starter cultures of *Lactobacillus bulgaricus* and *Streptococcus thermophilus* 1.1 (Danisco and Chr. Hansen, Denmark) were activated in skim milk at 37°C, and 2% of fresh and dry starter cultures was inoculated. The formulations were incubated at 43 ± 1°*C*, and the pH was monitored to about 4.5. Fortified yoghurt was cooled and stored overnight at 4°C. The plain stirred yoghurt products were packaged in tightly sealed containers and refrigerated at 4°C.

To perform a sensory evaluation, aliquots of yoghurt samples were flavored with four unique flavors: strawberry, passion, mango, and banana to mask the fishy flavor and taste.

### 2.8. Sensory Evaluation Analysis

#### 2.8.1. Sensory Evaluation of the Flavored Fortified Yoghurt Samples

A panel of 40 students, consisting of 22 males and 18 females with prior knowledge in aspects of the sensory evaluation, were selected from the Departments of Food Science and Nutrition and Dietetics of the Technical University of Kenya. The panel was first trained in descriptive sensory analysis. Before sensory profiling, descriptors for taste, aroma, color, flavor, mouthfeel, and after-taste were developed. The descriptors were evaluated on a structured five-point hedonic scale, and the samples were labeled using a three-digit code obtained from the table of random numbers. Potable water was used to rinse the mouth between samples. Data were collected using questionnaires for the various samples. The panel determined the most preferred flavor among strawberry, mango, passion, and banana.

#### 2.8.2. Determination of Quality Parameters in the Fortified Yoghurt

The extraction of lipids in the fortified yoghurt was performed as described by Robertson et al. [21]. To 5 g of the yoghurt sample, 5 ml distilled water and 30 ml 1 : 2 (*v*/*v*) CHCl_3_ : MeOH solution were added and vortexed. CHCl_3_ and dH_2_O (10 ml each) were added, respectively, and vortexed. Centrifugation (1000 × *g* at room temp) for 5 min yielded a two-phase system: an aqueous top layer and an organic bottom layer. To recover the organic phase, a Pasteur pipette was inserted through the upper phase with gentle positive pressure (gentle bubbling). The bottom layer was carefully withdrawn avoiding the interface or upper face.

The PV, AV, TOTOX, and FFA contents of the extracted lipids were determined as previously described.

### 2.9. Statistical Analysis

All statistical analyses were performed using SPSS software (IBM SPSS Statistics 19). The *post hoc* Tukey test was used for the sensory evaluation analysis. All experiments were performed in triplicate, and the means and standard deviations were reported.

## 3. Results

### 3.1. The Wet Weight Distribution of the Fish Oil Fractions

Enzymatic hydrolysis of fish pads yielded four phases: oily, emulsion, aqueous, and sludge phases. In the absence of an exogenous enzyme, only two phases were formed (oily and sludge phases). Omega-3-rich fish oils were recovered from the oily phase. An oil yield of 48.33 ± 1.15 g/100 g of the by-products was obtained in the absence of water in enzymatic hydrolysis (Table 2). However, oil recovery in the absence of Alcalase was higher at 60.67 ± 3.78 g/100 g (Table 2).

### 3.2. Recovery of Omega-3-Rich Fish Oils

Upon centrifugation of the hydrolase at 1000 × *g* for 5 min at room temperature and subjecting the recovered oily phase to a second centrifugation at 4 degrees, the recovered oil from enzymatic hydrolysis yielded 31.3 g of the liquid oil which was rich in omega-3 fatty acids and 10.1 g of saturated fatty acids. From 60.7 g of the oily phase without an exogenous enzyme, 48.42 g of the liquid fraction and 5.8 g of the solid fraction rich in saturated fatty acids were recovered.

### 3.3. PV, AV, TOTOX, and FFA

Quality parameters are indicators of initial lipid oxidation. Based on the study findings, the omega-3-rich fish oil quality parameters were within the acceptable range as shown in Table 3.

### 3.4. Acceptability of Differently Flavored Omega-3-Fortified Yoghurt

**Figure 1:**
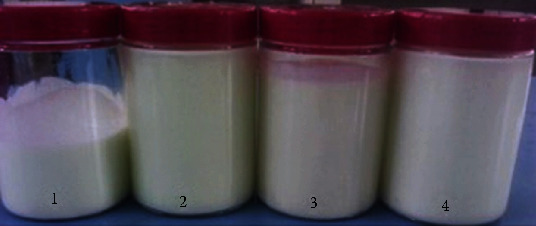
The four types of plain stirred fortified yoghurt. These included banana (1), passion (2), strawberry (3), and mango (4) flavors, respectively.

The four types of stirred fortified yoghurts produced are shown in Figure 1.

**Figure 2:**
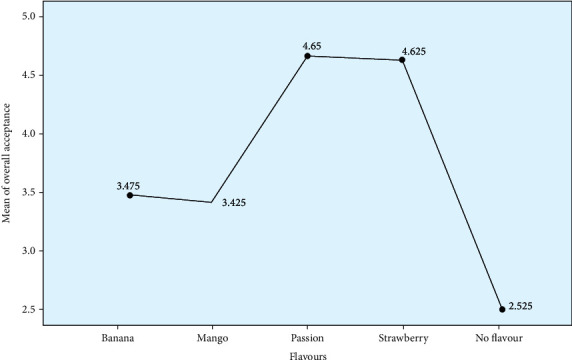
Means of overall acceptance of the four yoghurt flavors. Strawberry- and passion-flavored yoghurts were the most preferred.

Results from the 40 panelists who evaluated the four stirred fortified yoghurts indicated that passion and strawberry flavors were the most liked with mean values of 4.65 and 4.625, respectively, as shown in Figure 2.

The unflavored yoghurt sample was the most disliked and was in subset 1. There was no significant difference in the overall acceptance of mango and banana flavors and passion and strawberry flavors which were in the same subsets (*p* < 0.05). Overall, the panelists preferred strawberry and passion flavors whose scores were higher (4.63 and 4.65, respectively) compared to the rest (Table 4).

## 4. Discussion

Yoghurt is a milk product that has high nutritional value. Some of the essential nutritional benefits of consuming yoghurt include a reduction in the incidences of gastrointestinal illnesses, lactose intolerance, protection from pathogens due to the presence of active starter cultures, and antitumor and anticholesterol effects [22–24]. Increased yoghurt consumption has been attributed to alterations in plain yoghurt for desirable nutritional and healthy profiles, desirable texture, and unique flavors [23–25]. Because yoghurt is a popular food among adults and children, fortification of yoghurt with omega-3-rich sources has gained significant attention especially among health- and nutrition-conscious consumers [15]. Fish and other seafood are rich sources of omega-3 fatty acids. Nile perch is a warm water fish present in East African lakes and rivers, and its processing results in by-products such as fat pads that remain underexploited [26]. Local women purchase the fat pads and use them to deep fry fish since they are cheaper compared to other commercial fats and oils. Besides, there exists limited knowledge on the nutritional value of the fat pads [15, 27]. Previous studies have shown that the fat pads from viscera and belly flaps of Nile perch contained on average 750 mg (7.5%) of omega-3-rich fish oils from 100 g of Nile perch [16]. Another report also indicated that there were significant amounts of PUFAs in fish oil extracted from Lake Victoria Nile perch viscera [28].

In this study, the extraction of omega-3-rich fish oils from the fat pads in the presence and absence of enzyme gave an oil yield of 48% and 61%, respectively. The difference in the yields is attributed to the entrapment of oil in the emulsion phase that occurred in enzymatic hydrolysis. The emulsion consists of hydrophobic peptides associated with lipids [16]. The amount of oil recovered is affected by the proteins present in the raw material [9]. High protein content reduces the amount of oil recovered due to entrapped oil in the emulsion phase and further loss of much of the oil during the steps following hydrolysis.

This study, therefore, suggests that sufficient amounts of omega-3-rich fatty acids can be extracted from Nile perch fat pads in the absence of enzymes, which is cost-effective and efficient. Separation of the fish oils by TLC was achieved; this allowed an insight into the composition of the Nile perch fish oil. Based on the TLC separation, the fish oil comprised of TAGs, DAGs, MAGs, and FFA, and these were consistent with the findings reported by Mbatia et al. [16]. The PV, AV, TOTOX, and free fatty acid values were all within the acceptable range. The initial PV for all the samples was quite low, indicating that they were not oxidized. The hydrolysate had the highest PV value of 5.67 ± 0.76 meq/kg oil, whereas the oily and the emulsion phases had an AV of 4.83 ± 1.75 and 4.67 ± 1.26 meq/kg oil, respectively (Table 4). Fish oil with an AV of 5 meq/kg and below is considered fresh oil or one whose hydroperoxides are degraded into secondary oxidation products such as ketones and aldehydes [29]. The recommended AV for crude fish oil is 3-20 meq/kg [30]. The AV was determined to be 16.50 ± 2.14, 18.17 ± 1.88, and 20.81 ± 1.27 for the oil phase, fish protein hydrolysate, and the homogenate, respectively. This was an indication that the fish oils were not oxidized hence good for human consumption (Table 4). An AV of less than 20 has been set to determine the quality standards of the fish oil [30].

The homogenate had higher TOTOX values of 32.15 ± 1.42 compared to the oil and emulsion phases that had values of 26.16 ± 1.09 and 32.15 ± 1.42, respectively (Table 4). The FFA values for the three fractions were 2.16 ± 0.22, 1.55 ± 0.14, and 2.71 ± 0.43 for the oil phase, emulsion phase, and the homogenate, respectively (Table 4). The FFA content was below the recommended values of 3% in all the samples. FFA accumulates in the protein fraction and not in the oil fraction due to the polar groups hence the reduced FFA content in the oil fraction [28].

The use of a taste panel for sensory evaluation is one of the most accurate ways to determine the quality parameters of fish oil [31]. People can be trained to detect very low levels of volatile components that cannot be detected using traditional oxidation tests [28]. Fruit flavors are considered important in the acceptance of yoghurts. In this study, the panel was not made aware of the flavor system in the yoghurt to avoid a preconceived bias against the flavors. Therefore, based on the panel responses, the omega-3-fortified yoghurt is likely to be accepted by the greater general population, an indication that there is a potential market for the product especially among health- and nutrition-conscious consumers [10]. Omega-3 fatty acids from Nile perch by-products can also be used to enrich various food products such as margarine, fruit juices, milk, and sausages. This ensures that people consume the required omega-3 fatty acids through the diet hence achieve the associated health benefits [9].

## 5. Conclusion

The production of nutritionally superior yoghurt fortified with omega-3-rich fish oil extracted from Nile Perch by-products as a functional food was successfully achieved in this study. This yoghurt is desirable for general body health functions and in protection against cardiovascular diseases. Omega-3-fortified yoghurt delivers adequate heart-healthy omega-3 fatty acids to achieve the suggested daily intake of 150 mg/day through a single serving (150 g) of yoghurt. Fortification of yoghurt provides an alternative and easily incorporated source of omega-3 fatty acids. This innovation is vital for the nutrition- and health-conscious population, and hence, a potential market for this product exists.

## Figures and Tables

**Table 1:** Preparation of omega-3-fortified yoghurt.

Ingredient	YF	PY
Milk (l)	2	1
Skimmed milk powder (g)	80	20
Sugar (g)	—	—
Omega-3 fatty acid-rich oil (g)	53.3	—
*L. bulgaricus* and *S. thermophilus* (g)	0.5	0.25

YF: yoghurt fortified with fish oil; PY: plain natural yoghurt (control).

**Table 2:** Wet weight distribution of the fish oil fractions.

	Sample (with enzyme Alcalase) in grams	Control (g) without an enzyme
Oil	48.3 ± 1.15	60.7 ± 3.78
Emulsion	10.3 ± 0.58	—
Aqueous phase	2.7 ± 0.58	—
Sludge	2.0 ± 1.0	4.8 ± 1.96

Values are the means ± standard deviation.

**Table 3:** Quality parameters of the extracted fish oil fractions.

Parameter	Oil phase	Emulsion phase	Homogenate
PV (meq/kg)	4.83 ± 1.75	4.67 ± 1.26	5.67 ± 0.76
AV	16.50 ± 2.14	18.17 ± 1.88	20.81 ± 1.27
TOTOX	26.16 ± 1.09	27.51 ± 0.95	32.15 ± 1.42
FFA (%)	2.16 ± 0.22	27.51 ± 0.95	2.71 ± 0.43

Values presented are the mean ± SD of triplicates. PV: peroxide value; AV: anisidine value; TOTOX: total oxidation; FFA: free fatty acids.

**Table 4:** Homogeneous subsets of the different yoghurt flavors.

Overall acceptance				
Flavors	*N*	Subset for alpha = 0.05		
		1	2	3
No flavor	40	2.53		
Mango	40		3.43	
Banana	40		3.48	
Strawberry	40			4.63
Passion	40			4.65
Sig.		1.000	0.998	1.000

Means for the groups in homogeneous subsets are displayed.

## Data Availability

No datasets were generated or analyzed during the current study.
